# Equity in health care utilization in Chile

**DOI:** 10.1186/1475-9276-12-58

**Published:** 2013-08-12

**Authors:** Alicia Núñez, Chunhuei Chi

**Affiliations:** 1 Departamento de Control de Gestión y Sistemas de Información. Facultad de Economía y Negocios, Universidad de Chile, Diagonal Paraguay 257, oficina 1304, Santiago, Chile; 2 School of Biological and Population Health Science, Oregon State University, 013 Millam Hall, Corvallis, Oregon 97331-5109, USA

## Abstract

One of the most extensive Chilean health care reforms occurred in July 2005, when the Regime of Explicit Health Guarantees (AUGE) became effective. This reform guarantees coverage for a specific set of health conditions. Thus, the purpose of this study is to provide timely evidence for policy makers to understand the current distribution and equity of health care utilization in Chile.

The authors analyzed secondary data from the National Socioeconomic Survey (CASEN) for the years 1992–2009 and the 2006 Satisfaction and Out-of-Pocket Payment Survey to assess equity in health care utilization using two different approaches. First, we used a two-part model to estimate factors associated with the utilization of health care. Second, we decomposed income-related inequalities in medical care use into contributions of need and non-need factors and estimated a horizontal inequity index.

Findings of this empirical study include evidence of inequities in the Chilean health care system that are beneficial to the better-off. We also identified some key factors, including education and health care payment, which affect the utilization of health care services. Results of this study could help researchers and policy makers identify targets for improving equity in health care utilization and strengthening availability of health care services accordingly.

## Introduction

A high level of health status and a fair distribution of health care are both common goals that most societies and governments seek to pursue [1]. Guaranteeing access to health care services is one step that helps to preserve a good health status, which is also one of a government’s responsibilities, as stated in the World Health Organization Constitution [2]. Chile agrees with these objectives as well. In its national health objectives for the 2010–2020 period it includes, among others, living longer and better by increasing healthy longevity and reducing inequalities [3]. In Chile, health care coverage is provided through two parallel systems: the public and the private systems. Currently 70 per cent of the population is covered by the public fund and 17.5 per cent by private insurance [4]. The remaining population is covered by public institutions (military institutions, for example) and by not-for-profit organizations. Although health care is guaranteed to all Chileans regardless of their ability to pay, the duality of the system has led to an increase in inequalities in health care and overall population health. In response, a major health care system reform was passed in 2005 and implemented in 2006 to address growing inequalities. This reform established the coverage of sixty-nine health conditions that must be covered for free by the public and private system under a plan called AUGE, or regime with explicit health guarantees. The list of health conditions was established through prioritization, and using a progressive set of criteria, which includes burden of disease, effectiveness of the treatments, health care system capacity, financial burden and social consensus [5].

One of the great challenges for the country is to sustain and to improve the regime with an explicit health guarantee, which according to the World Bank is the first country in Latin America to legally establish principles of access, quality, opportunity, and financial protection [6]. There are pressures to increase the number of benefits included in AUGE with limited funding available.

To date, there has been very little empirical research on how well this reform has improved access to and the distribution of health care services in Chile, which, in turn motivated this study. This study seeks to fill a part of this gap in the literature by analyzing equity in health care utilization. That is, we intend to investigate this research question: Under the current Chilean health care system, are health care services equitably distributed? The importance of this investigation is that it not only seeks to measure the existence of inequity, but also enables comparison across time periods and identifies factors that affect equity. Findings from this empirical study will provide timely evidence for policy makers to understand the current distribution and equity of health care utilization, and strengthen the availability and equity of health care services accordingly.

In the Background section of this paper briefly describes the Chilean health care system. In Data section discusses the data used for this study and the methods for the two-part model estimation for total health care use and also for specific individual health care services such as the use of preventive care, general practitioner visits, specialty care, and emergency care. It also includes discussions of the methods used to assess horizontal equity in health care utilization. In Research Method section provides the definitions of key variables and their summary statistics. The main empirical results are reported in Definition of key variables section. The last section contains the main conclusions drawn from this study.

## Background

### Chilean health care system

Chile is an upper-middle income country located in South America. Chile had the fastest-growing economy in Latin America in the 1990s and has weathered recent regional economic instability. Among Latin American and income comparable countries, Chile exhibits a categorically high level of health indicators; average life expectancy at birth in 2009 for men was 76 years compared to the regional average of 73 years [7]. Likewise, average life expectancy at birth for women was 82 years compared to the regional average of 79 years. The under-five mortality rate for both sexes was 9 per 1,000 in comparison to 18 per 1,000 at the regional level [7]. In addition to these good health indicators, health conditions have changed in the past 50 years [8]. The percentage of malnutrition among children aged less than 6 years decreased from 37% to 2.9% in 40 years (1960–2000). However, obesity among 4-year-old children in a recent study is approaching 20% [8]. Thus, there are series of lifestyle changes that the country still has to face; such as improving balance of food consumption, increasing physical activity, reducing tobacco consumption, reducing stress level [9], together with socioeconomic challenges, which also include changes in the copper-dependent economy, and the continued unequal distribution of wealth.

The Chilean health care system is a dual system, which is publicly and privately financed. There is a single public insurance (FONASA) and several private health insurance companies (ISAPREs). FONASA cover more than 11 million beneficiaries, approximately 70 per cent of the Chilean population [4]. ISAPREs provide services to approximately 17.5 per cent of the Chilean population, and other forms of government insurances such as the National Defense Pension Fund and the National Insurance for the Police, represented around 10 per cent of the population [4]. The FONASA and ISAPREs systems share a common funding source coming from the obligatory contribution of employees, which is a 7 per cent income tax with a limit of 60 “unidades de fomento” (UF, unit of account, used in Chile for calculating monetary principles and interests in internationally secured loans for development), equivalent to US$2,852.50 dollars [10]. Workers can choose to be covered either by FONASA or one of the seven ISAPREs operating in the country [11].

FONASA completely or partially covers those people with limited resources. It is structured in 4 groups classified by income: people in groups A and B (lower income groups) receive free health care services, group C have to pay 10% cost-sharing, and group D pay 20% of health services [12]. Except for Group A, the rest have the option of using the Free Choice Scheme, which consists of the utilization of private health care subscribed by FONASA through the purchase of bonds. Under the Free Choice Scheme the co-payment levels will vary according to the insurance plan and the medical services received. On the other hand, ISAPREs can offer different premiums to their customers to improve their health plans. In relation to pharmaceuticals, both FONASA and ISAPRES include intra-hospital medication. In the public system, primary care medicines do not require copayment. Otherwise, the copayment will vary according to the group or insurance plan [13].

### Equity, need, and access

#### Equity

There is a broad consensus on the need to measure health equity, but there is a considerable disagreement in the definitions and the measurement of equity [14-18]. The World Health Organization (WHO) defines inequity as “differences [in health status], which are unnecessary and avoidable, but in addition are considered unfair and unjust.” This definition, however, still leaves open how one defines “unfair” or “unjust”.

One notion of health equity that has reached a broad consensus among many health policy makers and researchers is the idea of allocating health services according to need as opposed to willingness or ability to pay for services [19]. In this study we employ this widely used approach to define equity into two dimensions: horizontal and vertical equity. Horizontal equity implies that people with equal need receive equal treatment. On the other hand, vertical equity is when individuals with unequal need receive unequal treatments. Further, we can assess equity in multiple ways by using concepts of health status, distribution of resources, expenditures, utilization, and access [16].

#### Access

Access refers to the level of health care services that the system is able to provide to an individual. Access has often been measured by proxy measurements such as the use of health care services depending on the need for care [20]. However, access entails a broader set of concerns and dimensions about how individuals are going to satisfy their health care need. Pioneer researchers in access such as Le Grand [21] and Mooney [22] have argued that access and receipt of treatment are different concepts. Access to treatment refers to the opportunity open to people to receive health care treatment, while receipt of treatment reflects the opportunity and also the actual use of health care treatments [16]. A definition of access that takes into account this situation is one that defines access as the maximum level of care that a person can consume, given their income, and the time and cost associated with the health care consumed [23].

Access is a complex health policy concept; and it becomes a challenge to measure it in a more comprehensive way [24]. In order to measure it, some researchers use the utilization of health care services as a proxy variable while others have proposed to evaluate access according to characteristics of the population such as insurance coverage or family income. Furthermore, some researchers use the characteristics of the delivery system as a measurement for access such as the distribution and availability of health care facilities [25].

In this study, definition of access was focused primarily on financial dimension, including health insurance. Other dimensions of access were not included in this analysis, partly due to data limitation. We approximate access by including two main components; the use of health services, and factors that facilitate or impede the use of health care services. We are aware that measuring utilization considers only one part of the population, i.e. those who are already using medical services. Having access to health care services does not mean that one actually use medical care. This study focuses on health care utilization as a proxy measure of access, i.e. individuals who used the health care services during a year. As such, we recognized the limitation of using utilization as a proxy measurement being incomplete to represent the concept of access.

#### Need

This research follows an egalitarian principle, which means that health care should be distributed according to a need principle rather than people’s willingness and ability to pay, which is the free market principle. Egalitarians claim that an appropriate allocation of medical care according to need promotes health equality [26]. However, Culyer and Wagstaff [16] have shown that allocating health care expenditures according to need does not necessarily result in or promote equality of health. Instead, it will depend on the definition of need adopted.

One of the most popular definitions establishes that need measures the care that is required to obtain the maximum possible health improvement within given resource constraints [27]. Therefore, unmet needs arise from the absence of care when resources were available. In this study, the indicators used in the prediction of needed health care are demographic variables (age-sex dummy variables) plus health status and morbidity variables (self-reported health problems and presence of chronic conditions).

## Data

The data for this study are drawn from the National Socioeconomic Survey (CASEN, 1992–2009) and the 2006 Satisfaction and Out-of-Pocket Payment Survey. Both surveys have national coverage. CASEN is a biannual household survey that represents the national, regional, urban, and rural areas in Chile [28]. The survey has been carried out since 1985; however, we are limiting our analysis to surveys starting from 1992 because of changes in the CASEN questionnaires. In this way we can maintain consistency in the variables that are analyzed. The main purpose of the survey is to describe the socioeconomic situation in Chile. It also includes a section on health status. The sampling method is multi-stage random sampling with geographical stratification and clustering. The final sample for each year is close to 65,000 households and 260,000 people, where the share of men and women in the sample is 49% and 51% respectively.

The first National Survey on Satisfaction and Out-of-Pocket Payment was carried out in 2006, and there are no new versions of this survey to date [29]. It represents national and urban households. The sample design is strictly probabilistic, multistage (5 stages), geographical stratification and conglomerates. The sample of the survey involves 4,558 households or 16,519 individuals.

## Research method

Using these two surveys, we assess equity in the use of health care services by two approaches. First, we estimated a two-part model based on the cross-sectional data from the CASEN survey and the 2006 Satisfaction and Out-of-Pocket Payment data. For the two-part model, we first estimated a logit model, where the dependent variable is a binary variable of whether the respondent used health care services. Health care utilization data is known to have a skewed distribution with many of the people surveyed having no health care use in the recall period. Therefore, a logit model is more appropriate given the distribution of the data, which is highly concentrated in the tails. The results from the logit model will contribute to the second stage of the analysis. In the second part of the model we estimated a linear regression model for the frequency of health care use. Finally, we decomposed income-related inequalities in medical care use into contributions of need and non-need factors and estimated a horizontal inequity index using the 2006 Satisfaction and Out-of-Pocket Payment data. The purpose of including the estimation of horizontal equity index analysis is to provide supplemental information for our equity analysis. While the two-part model estimation can provide information on whether and to what extent social-economic factors affect access to health care, estimated horizontal equity index can help put our two-part model analysis in a broad perspective of how equitable access to health care was distributed in the society as a whole.

### Two-part model

To better understand equity in health care use, it is imperative to identify factors associated with its use and assess the extent to which these factors may contribute to inequities in the system. In order to identify such factors we estimated two-part models not only for examining health care utilization at an aggregate level but we also estimated separate models in the following categories of health care services: preventive care, general practitioner visits, specialty visits, and emergency care. To implement this analysis, we included the data collected by the CASEN survey for the years 1992 to 2009. We decided to use the two-part model for evaluating health care utilization because the decision on whether to use health care services and the quantity of use is based on a two-step process. Generally, the initial visit to a physician or health service provider depends largely on the patient, while the following visits are associated with other factors such as the quality and satisfaction of the services, and the influence of the physician, among others. A measure of health care utilization like this, which includes the number of visits to physicians over a given period of time, is a discrete and non-negative value count. We have datasets with a large proportion of zeros, representing those who did not receive health care services during the recall period of data collection. Zero counts and positive counts in health care utilization represent the actual level of use of medical services. A zero value does not represent a missing value, and they are required in order to understand the level of use of medical services [30].

Conceptually, the two-part model can solve the problem of excess of zeros and is a more appropriate model than using negative binomial or Poisson models [31]. According to Gerdtham [32], Polhlmeier and Ulrich [33] two-part models provide a better fit to health care utilization than negative binomial or Poisson models. We recognize that there is a debate on which model is more appropriate to handle the excess of zeros, either the two-part model or zero-inflated models, such as the zero-inflated Poisson and the zero-negative binomial. More details on such debate can be found in Jones [34].

The dependent variable for the two-part model was the number of self-reported health care visits during a year. In order to estimate the model we constructed two variables. The first is a dichotomous variable indicating the use or non-use of health care services; the second variable indicates the number of health care visits. We repeat this model to analyze the other four types of health services under study, which are preventive visits, general practitioner visits, specialty visits, and emergency visits. The main explanatory variables for the CASEN dataset were geographical region, gender, age, marital status, availability of electricity, water and waste disposal, type of housing and housing ownership status, education level, insurance system, working hours, income, and health care payment.

We also used the 2006 Satisfaction and Out-of Pocket data to carry out a two-part model estimation. The analysis was conducted at the individual level since the two-part model is well suited to model individual level health care utilization data. This model allows us to address issues that cannot be addressed at the aggregate level like assessing separate effects of key variables on health care utilization among all individuals. The main explanatory variables were geographical region, gender, civil status, education level, work status, chronic disease, accidents, type of housing and housing ownership status, type of insurance, beneficiaries from the insurance system, dependent worker, emergency insurance, additional insurance, debt, work insurance, total number of people in the household, health care expenditures, income, health care satisfaction, and AUGE. We used STATA 11 for our model estimations.

### Equity in health care utilization

A widely used method to estimate equity is measuring it through the analysis of need factors, and whether factors other than need affect the utilization of health care. However, need is a concept that can be difficult to define and measure [27]. In this study we relied on demographics and health conditions as a proxy measured for need.

We use the indirect method comparing the differences between actual need and need-standardized distributions for the probability of using health care during a year. As suggested by O’Donnel et al. [35], we use non-linear estimation to include the large proportion of observations with no utilization of health care services. We specified a probit model with control variables to show the difference between need-predicted use and actual use [35]. Also, we computed need-standardized health care use with and without controls using ordinary least square (OLS) and probit models. In these models, we use a set of eighteen dummy variables representing health care problems from the 2006 Satisfaction and Out-of-Pocket Payment survey, which include: hypertension, diabetes, heart disease, respiratory disease, loss of vision, dental problems, depression, stomach problems, gynecological problems, fever, accident, arthritis, headache, pregnancy, a bug, circulatory problems, and other problems in the population, and seven age-sex dummies as a proxy of need, with four age groups (0–24, 25–49, 50–74, 75+) for each gender.

### Indirect standardization using OLS

We use the method proposed by O’Donnel et al. [35] where utilization can be defined by a linear regression model as shown in equation 1:

(1) $$y_{1}\;=\;\alpha \;+\;\beta \ln\left(\mathrm{in}c_{i}\right)+\sum_{j}\;\beta_{j}x_{\mathrm{ji}}+\sum_{k}\gamma_{k}z_{\mathrm{ki}}+\epsilon_{i}$$

where ln(inc_i_) is the natural logarithm of income, x_j_ are the proxy variables of need, and z_k_ are the non-need control variables.

We can compute the predicted or x-expected values using equation 2:

(2) $${\hat{y}}_{i}^{x}\;=\;\alpha +\beta \mathrm{lnin}c_{i}+\sum_{j}\;{\hat{\beta }}_{j}x_{\mathrm{ji}}+\sum_{k}{\hat{\gamma }}_{k}{\bar{z}}_{k}$$

Finally, we obtained the indirect need standardized utilization as shown by equation 3:

(3) $$j_{i}^{\mathrm{IS}}\;=\;y_{i}\;-\;{\hat{y}}_{i}^{X}+\bar{y}$$

Indirect standardization using nonlinear models:

For the nonlinear model, health care utilization is defined by equation 4:

(4) $$y_{i}\;=\;G\left(\alpha +\sum_{j}\;\beta_{j}x_{\mathrm{ji}}+\sum_{k}\gamma_{k}z_{\mathrm{ki}}\right)+\epsilon_{i}$$

The functional form G can take different forms for probit, logit or other model. In this study we use a logit model.

Finally, we standardized need as shown by equation 5:

(5) $$\begin{array}{l} {\hat{y}}_{i}^{\mathrm{IS}}\;=y_{i}\;-\;G\left(\hat{\alpha }+\sum_{j}\;{\hat{\beta }}_{j}x_{\mathrm{ji}}+\sum_{k}{\hat{\gamma }}_{k}{\bar{z}}_{k}\right)\\ \;+\frac{1}{n}\sum_{i=1}^{n}G\left(\hat{a}+\sum_{j}\;{\hat{\beta }}_{j}x_{\mathrm{ji}}+\sum_{k}\gamma_{k}{\bar{z}}_{k}\right) \end{array}$$

### 4.3. Horizontal equity

We use a concentration index as a measure of income-related inequality in the use of health care. According to Wagstaff and van Doorslaer [26] horizontal inequity is measured by comparing actual utilization of medical services L_M_(p) with the need concentration curve L_N_(p). Horizontal inequity, therefore, is defined as twice the area between the need and medical care concentration curve. C_M_ denotes the concentration index for actual use of health care and C_N_ indicates concentration curve for needed use of health care, as indicated in equation 6. When HI_wv_ takes a positive value, horizontal inequity is favoring the economically better-off; in contrast, if HI_wv_ takes a negative value, horizontal inequity is favoring the economically worse-off. A zero value indicates that need and medical care are proportional, regardless of income [26].

(6) HIWV=s∫01LNp−Lmpdp=Cm−Cn

As it has been established health concentration index can be decomposed into the contribution of explanatory factors (see equation 7). Each factor’s contribution is the product of the elasticity of health status with respect to the specific factor and the level of income-related inequality in that factor [35-37].

(7) $$y_{i}\;=\;\alpha +\sum_{k}\beta_{k}x_{\mathrm{ki}}+\epsilon_{i}$$

In equation 7 *y*_*i*_, is health care use for an individual *i*, x is the determinants of health care and ϵ is the disturbance term. Then the concentration index can be written as equation 8, which is indicating that the concentration index for health is a weighted sum of the concentration indexes of the different determinants of health and a residual term or inequality that cannot be explained by systematic variation across income groups, as expressed in equation 8.

(8) $$C_{M}\;=\sum_{k}\left(\beta_{k}{\bar{x}}_{k}/\bar{y}\right)C_{k}+GC_{\epsilon }/\bar{y}$$

In equation 8, $\bar{y}$ is the mean of y, ${\bar{x}}_{k}$ is the mean of *x*_*k*_, C_k_ is the concentration index for each health determinant (*x*_*k*_) and the generalized concentration index for the disturbance term is *GC*_*k*_.

Using this method, we are able to measure the contribution of each factor and also the importance within the total contribution to inequality in health care use.

In a linear model, we can use equation 9 to decompose inequality in health care use into:

(a) direct contribution of income, which is the product of the income elasticity on health care use and the concentration index of income;

(b) the contribution of need variables;

(c) the contribution of non-need variables; and

(d) residual term.

An alternative estimate of horizontal inequity is to take the concentration index of health care use minus (a), (b), and (c).

(9) $$\begin{array}{l} C\;=\;\underset{a}{\underset{︸}{\left(\beta_{r}{\bar{x}}_{r}/\bar{y}\right){\hat{C}}_{r}}}\underset{b}{\underset{︸}{+\;\sum_{n}\left(\beta_{n}{\bar{x}}_{n}/\bar{y}\right){\hat{C}}_{r}}}\\ \;\underset{c}{\underset{︸}{+\;\sum_{p}\left(\beta_{p}{\bar{x}}_{p}/\bar{y}\right){\hat{C}}_{p}}}\underset{d}{\underset{︸}{+\;GC_{\epsilon }/\bar{y}}} \end{array}$$

where ${\bar{x}}_{r}$ is the mean of income itself, ${\bar{x}}_{n}$ is the contribution of need standardizing variables, and ${\bar{x}}_{p}$ the contribution of other non-need variables.

This method for decomposition holds only if we are working with linear regressions [37]. We need to use a nonlinear approximation in the case of nonlinear models, such as health care use when there is a large number of people who did not use health care services during the recall period.

The general form of a nonlinear regression is given by equation 4. Similar to the linear case, we need to compute the need-expected utilization, which is defined by equation 10.

(10) $$y_{i}\;=\;G\left(\sum_{n}\;{\hat{\beta }}_{n}x_{i}^{n}+\sum_{r}{\hat{\beta }}_{r}{\bar{x}}_{i}^{r}+\sum_{p}{\hat{\beta }}_{p}{\bar{x}}_{i}^{p}\right)$$

where *x*^*n*^ represents need-expected utilization, ${\bar{x}}^{r}$ is the mean of income and ${\bar{x}}^{p}$ is the mean for other non-need variables. It should however be noted that in a nonlinear setting the decomposition requires a linear approximation [37]. We applied a linear approximation, and present it in equation 11.

(11) $$y_{i}\;=\;\alpha^{m}+\sum_{j}\;\beta_{j}^{m}x_{\mathrm{ji}}+\sum_{k}\gamma_{k}^{m}z_{\mathrm{ki}}+u_{i}$$

In equation 11, $\beta_{j}^{m}$ and $\gamma_{k}^{m}$ are partial effects for the need (x) and control (z) variables, and u is the error term. Using this approximation, we are able to finally compute the decomposition result C_M_[38] as equation 12:

(12) $$C_{m}\;=\;\sum_{j}\left(\beta_{{}^{j}}^{m}{\bar{x}}_{j}/\mu \right)C_{j}+\sum_{k}\left(\gamma_{k}^{m}{\bar{z}}_{k}/\mu \right)C_{k}+GC_{\mu }/\mu$$

where μ is the mean of y, ${\bar{x}}_{j}$ is the mean of *x*_*j*_, and ${\bar{z}}_{k}$ is the mean of *z*_*k*_.

## Definition of key variables

Table 1 displays the variables selected from the National Socioeconomic Survey (CASEN) for our model estimation for the years 1992–2009.

**Table 1:** **Variables selected for this study from the National Socioeconomic Survey** (**CASEN**, **1992**–**2009**)

**Variable**	**Measurement scale**	**Definition**
Health care use	Nominal/Discrete	Health care use: 1 = yes; 0 = no
Region	Nominal/Discrete	Live in a different region than the metropolitan region (1 to 13)
Number_house	Ratio/Discrete	Number of people in the family (0 to 10)
Gender	Nominal/Discrete	Gender: 0 = male and 1 = female
Age	Interval/Discrete	Age in years at the time of the survey (0 to 110)
Married	Nominal/Discrete	Married marital status: 1 = yes, 0 = no
Electricity	Nominal/Discrete	Availability of electricity: 1 = yes; 0 = no
Water	Nominal/Discrete	Availability of water: 1 = yes; 0 = no
Disposal	Nominal/Discrete	Availability of waste disposal: 1 = yes; 0 = no
Housing	Nominal/Discrete	Type of housing: 1 = house, 2 = apartment, 3 = tenement, 4 = hut/shack, 5 = other, 6 = room
House	Nominal/Discrete	Residential status? 1 = own, 2 = rent, 3 = transfer, 4 = illegal occupation, 5 = other
Education	Nominal/Discrete	Ever attending or currently attending to school?: 0 = no, 1 = yes
Insurance	Nominal/Discrete	Do you belong to an insurance system? 1 = public system (indigent, group a) 2 = public system (group b), 3 = public system (group c), 4 = public system (group d), 5 = public system (do not know the group), 6 = armed forces, 7 = Isapres, 8 = private, 9 = other system,
Hours	Ratio/Discrete	Working hours per week
Ln(income)	Ratio/Continuous	Natural logarithm of income
NHC	Ratio/Discrete	Number of health care visits
Pay	Nominal/Discrete	Out-of-pocket health care payment: pay, sometimes pay, other type of payment, do not pay
Prevcare	Ratio/Discrete	Number of preventive care visits (0 to 90)
Attention	Ratio/Discrete	Number of doctor’s office visits (0 to 90)
Specialty	Ratio/Discrete	Number of specialty visits (0 to 90)
Emergency	Ratio/Discrete	Number of emergency visits (0 to 40)
Ethnicity	Nominal/Discrete	0 = none, 1 = aymara, 2 = rapa-nui, 3 = quechua, 4 = mapuche, 5 = atacameno, 6 = coya, 7 = kawaskar, 8 = yagan, 9 = no data
Health status	Ordinal/Discrete	Health status? 1 = very good/good, 2 = regular, 3 = very bad/bad, 4 = no answer
AUGE	Nominal/Discrete	Was the care you received covered by AUGE? 1 = yes; o = no

The response variable for the logit model is a dichotomous variable for health care use, referring to whether or not people have had any utilization of health care services during the period of analysis. The response variable for the OLS model is the frequency of health care use, excluding those who did not have any usage. In order to be consistent with the method of decomposing the concentration index, explanatory variables in the regression model are classified into three groups: income, need variables and other variables. Need variables include gender, age, and health status. Other variables include geographical region, marital status, and availability of basic services such as electricity and water, type of housing, level of education, health insurance, health care payment, and AUGE coverage.

Table 2 shows the variables selected from the 2006 Satisfaction and Out-of-Pocket payment survey for our model estimation.

**Table 2:** **Variables from the 2006 Satisfaction and Out**-**of**-**Pocket Payment Survey**

**Variable**	**Measurement scale**	**Definition**
Health care	Nominal/Discrete	Health care visits: 0 = no, 1 = yes
Age	Interval/Discrete	Age in years at the time of the survey (18 to 98)
Gender	Nominal/Discrete	Gender: 0 = male and 1 = female
Civil status	Nominal/Discrete	Civil status: married, living in-partner, annulled/separated/divorce, widowed, and single
School	Ordinal/Discrete	Last level of study approved: no education, elementary school, high school, technical-professional school, technical training center, professional institute, university
Region	Nominal/Discrete	II region, V region, VIII region or XIII region
Work status	Nominal/Discrete	Did you work last week? 0 = no, 1 = yes
Chronic Disease	Nominal/Discrete	Do you have a chronic disease? 0 = no, 1 = yes
Accident	Nominal/Discrete	Did you have an accident? 0 = no, 1 = yes
Housing	Nominal/Discrete	Type of housing: 1 = house, 2 = apartment, 3 = tenement, 4 = hut/shack
House	Nominal/Discrete	Residential status? 1 = own, 2 = rent, 3 = transfer
Ln(income)	Interval/Continuous	Individuals level of income (0 to 15.90)
Insurance	Nominal/Discrete	Type of insurance system: Indigent card, Fonasa, Isapre, Capredena, Dipreca, other system, no insurance
Beneficiaries	Interval/Discrete	Number of people who can use the insurance coverage
Dependent	Nominal/Discrete	Are you a dependent worker whose employer automatically deducts the payment for health insurance? 0 = no, 1 = yes
Additional benefits	Nominal/Discrete	Do you pay more than the required 7 % of your taxable salary for health insurance? 1 = yes, 2 = no, 3 = does not apply
Additional insurance	Nominal/Discrete	Do you have additional insurance? 0 = no, 1 = yes
Emergency insurance	Nominal/Discrete	Do you have emergency insurance? 0 = no, 1 = yes
Debt	Nominal/Discrete	During the past year have you had to borrow money to pay health care costs? 0 = no, 1 = yes
Work insurance	Nominal/Discrete	Are you receiving welfare or social security? 0 = no, 1 = yes
Number of people in the household	Interval/Discrete	Total number of people in the household (1 to 18)
Ln(OOP)	Interval/Continuous	Natural logarithm of out-of-pocket payment (0 to 13.31)
Health care satisfaction	Ordinal/Discrete	Overall health care satisfaction? 1 = very satisfied/satisfied, 2 = indifferent, 3 = very dissatisfied/dissatisfied, 4 = no answer
AUGE	Nominal/Discrete	Was your health problem covered by AUGE? 1 = No AUGE pathology, 2 = AUGE pathology, 3 = do not know, 4 = no answer

Similar to Table 2 the response variable for the logit model is a dichotomous indicator of whether a person has had health care utilization during a particular year or not. The response variable for the OLS model is the frequency of health care use. We also organized the variables into three groups: income, need variables and other variables. Need variables include age, gender and health status. In this survey health status is self-reported, which include chronic diseases and accidents. Other variables included in the model are civil status, region, education level, work status, type of housing, health insurance, health care satisfaction, and AUGE coverage.

In addition, explanatory variables such as out-of-pocket payment and insurance may be endogenous. To test the endogeneity of some of the explanatory variables, we utilized a version of the Durbin-Wu-Hausman test using instrumental variables. We concluded at the five per cent level that the null hypothesis that the variables are exogenous cannot be rejected. We also performed likelihood ratios to assess the fit of our model and omitted variables.

## Results

### Two-part model for the CASEN survey

We use a two-part model to assess which factors are playing an important role in explaining inequity in health care use. We specified the two-part model based on both empirical literature and our understanding of the Chilean health care system, and tested several models, rather than a step-wise approach. The final model have been subjected to a number of specification and diagnostic tests, and presented the best model. Table 3 presents the estimation results of the two-part model using the data from the CASEN survey during 1992–2009.

**Table 3:** **Estimation results of two**-**part model for health care use from CASEN survey during 1992**-**2009**

**Variable**	**Logit model**	**OLS (Health Care Use)**	**OLS (Preventive Care)**	**OLS (Practitioner Visit)**	**OLS (Specialty Visits)**	**OLS (Emergency Visits)**
Year						
1992	(base)	(base)	(base)	(base)	(base)	(base)
1994	−0.121 (-14.44) ^***^	−0.129 (-7.72) ^***^	−0.073 (-7.51) ^***^	−0.058 (-6.74) ^***^	−0.041 (-4.85) ^***^	0.043 (8.63) ^***^
1996	−0.342 (-35.42) ^***^	−0.086 (-4.30) ^***^	−0.135 (-11.68) ^***^	0.049 (4.82) ^***^	−0.014 (-1.42)	0.015 (2.51) ^**^
1998	−0.201 (-23.12) ^***^	−0.056 (-3.18) ^***^	−0.157 (-15.37) ^***^	0.022 (2.50) ^**^	0.025 (2.89) ^***^	0.054 (10.38) ^***^
2000	0.300 (39.95) ^***^	−0.031 (-2.14) ^**^	0.025 (2.92) ^***^	−0.210 (-27.91) ^***^	0.100 (13.58) ^***^	0.054 (12.51) ^***^
2003	0.366 (48.40) ^***^	−0.081 (-5.51) ^***^	−0.031 (-3.65) ^***^	−0.237 (-31.38) ^***^	0.108 (14.70) ^***^	0.079 (18.20) ^***^
2006	0.281 (36.54) ^***^	0.368 (24.40) ^***^	0.076 (8.67) ^***^	0.084 (10.82) ^***^	0.072 (9.60) ^***^	0.136 (30.66) ^***^
2009	−0.140 (-17.04) ^***^	0.037 (2.33) ^**^	−0.059 (-6.23) ^***^	−0.027 (-3.27) ^***^	0.009 (1.14)	0.115 (24.06) ^***^
Region:						
I	(base)	(base)	(base)	(base)	(base)	(base)
II	0.037 (2.56) ^***^	0.140 (5.06) ^***^	−0.022 (-1.37)	0.053 (3.94) ^***^	0.063 (4.54) ^***^	0.044 (5.35) ^***^
III	0.055 (3.80) ^***^	0.167 (5.95) ^***^	0.054 (3.30) ^***^	0.050 (3.48) ^***^	0.021 (1.49)	0.042 (5.11) ^***^
IV	0.184 (14.16) ^***^	0.036 (1.46)	−0.012 (-0.85)	0.027 (2.09) ^**^	−0.006 (-0.46)	0.028 (3.78) ^***^
V	0.160 (13.80) ^***^	0.127 (5.71) ^***^	−0.002 (-0.17)	0.015 (1.32)	0.044 (3.98) ^***^	0.070 (10.68) ^***^
VI	0.043 (3.52) ^***^	0.136 (5.70) ^***^	0.061 (4.38) ^***^	0.042 (3.42) ^***^	−0.006 (-0.49)	0.039 (5.60) ^***^
VII	0.076 (6.40) ^***^	0.073 (3.18) ^***^	0.060 (4.45) ^***^	0.011 (0.90)	−0.010 (-0.87)	0.013 (1.89) ^*^
VIII	0.184 (16.51) ^***^	0.111 (5.17) ^***^	0.017 (1.36)	0.027 (2.37) ^**^	0.026 (2.37) ^**^	0.042 (6.71) ^***^
IX	0.196 (16.30) ^***^	−0.002 (-0.08)	−0.086 (-6.42) ^***^	0.037 (3.10) ^***^	−0.030 (-2.57) ^***^	0.078 (6.71) ^***^
X	0.082 (6.88) ^***^	−0.054 (-2.35) ^**^	−0.091 (-6.80) ^***^	0.019 (1.59)	−0.028 (-2.46) ^**^	0.046 (6.87) ^***^
XI	−0.009 (-0.47)	0.094 (2.69) ^***^	−0.067 (-3.26) ^***^	0.112 (6.24) ^***^	−0.014 (-0.82)	0.063 (6.10) ^***^
XII	0.068 (3.62) ^***^	0.119 (3.31) ^***^	−0.055 (-2.60) ^***^	0.076 (4.09) ^***^	0.032 (1.76) ^*^	0.067 (6.31) ^***^
XIII	0.109 (9.94) ^***^	0.180 (8.48) ^***^	−0.027 (-2.19) ^**^	0.064 (5.85) ^***^	0.104 (9.84) ^***^	0.039 (6.26) ^***^
Number_house	−0.026 (-5.95) ^***^	0.001 (0.15)	0.089 (17.61) ^***^	−0.053 (-11.86) ^***^	−0.016 (-3.69) ^***^	−0.019 (-7.44) ^***^
Gender	0.408 (110.58) ^***^	0.069 (9.90) ^***^	0.069 (16.98) ^***^	0.001 (0.29)	0.021 (6.05) ^***^	−0.022 (-10.80) ^***^
Age	0.008 (76.58) ^***^	0.014 (84.91) ^***^	0.009 (87.16) ^***^	0.004 (47.02) ^***^	0.002 (23.82) ^***^	−0.0003 (-6.44) ^***^
Married	0.118 (27.34) ^***^	0.003 (0.36)	0.065 (14.02) ^***^	−0.035 (-8.45) ^***^	0.002 (0.53)	−0.030 (-12.76) ^***^
Electricity	0.153 (15.52) ^***^	0.108 (5.55) ^***^	0.030 (2.69) ^***^	0.011 (1.12)	0.028 (2.90) ^***^	0.038 (6.65) ^***^
Water	0.147 (19.73) ^***^	0.098 (6.80) ^***^	0.034 (4.07) ^***^	−0.004 (-0.56)	0.031 (4.35) ^***^	0.037 (8.64) ^***^
Disposal	−0.088 (-8.23) ^***^	−0.095 (-4.64) ^***^	−0.025 (-2.14) ^**^	−0.032 (-3.05) ^***^	−0.018 (-1.75) ^*^	−0.020 (-3.26) ^***^
Housing:						
House	(base)	(base)	(base)	(base)	(base)	(base)
Apartment	0.172 (16.68) ^***^	0.132 (6.96) ^***^	0.017 (1.50)	0.007 (0.77)	0.089 (9.37) ^***^	0.019 (3.44) ^***^
Tenement	0.155 (4.42) ^***^	0.142 (2.15) ^**^	−0.043 (-1.12)	0.074 (2.18) ^**^	0.058 (1.75) ^*^	0.054 (2.78) ^***^
Hut/shack	0.136 (13.25) ^***^	0.046 (2.37) ^**^	0.019 (1.65) ^*^	0.004 (0.38)	0.001 (0.09)	0.023 (3.98) ^***^
Other	0.102 (1.56)	−0.196 (-1.59)	−0.111 (-1.55)	−0.057 (-0.90)	−0.015 (-0.24)	−0.013 (-0.35)
Room	0.103 (3.28) ^***^	0.119 (1.96) ^**^	0.138 (3.89) ^***^	−0.028 (-0.90)	−0.003 (-0.09)	0.012 (0.67)
House:						
Own	(base)	(base)	(base)	(base)	(base)	(base)
Rent	0.159 (27.73) ^***^	0.080 (7.39) ^***^	0.031 (4.90) ^***^	0.0001 (0.03)	0.017 (3.21) ^***^	0.032 (9.93) ^***^
Transfer	0.087 (17.46) ^***^	−0.012 (-1.27)	−0.0003 (-0.06)	−0.011 (-2.30) ^**^	−0.004 (-0.95)	0.004 (1.42)
Illegal occupation	0.025 (0.93)	0.005 (0.09)	0.008 (0.25)	−0.015 (-0.54)	0.003 (0.10)	0.009 (0.58)
other	0.166 (9.94) ^***^	0.041 (1.23)	0.078 (4.07) ^***^	−0.028 (-1.68) ^*^	0.011 (0.68)	−0.020 (-2.10) ^**^
School	−1.188 (-202.99) ^***^	−0.520 (-54.59) ^***^	−0.662 (-119.36) ^***^	0.048 (9.81) ^***^	0.055 (11.50) ^***^	0.040 (14.26) ^***^
Insurance	0.178 (46.03) ^***^	0.012 (1.54)	0.008 (1.81) ^*^	−0.004 (-1.11)	0.018 (4.73) ^***^	−0.010 (-4.45) ^***^
Working hours	−0.013 (-145.42) ^***^	−0.005 (-27.43) ^***^	−0.005 (-48.65) ^***^	0.0002 (2.75) ^***^	−0.001 (-7.39) ^***^	0.001 (10.90) ^***^
Ln(income)	0.178 (80.53) ^***^	−0.006 (-1.35)	0.023 (9.15) ^***^	−0.031 (-13.76) ^***^	0.023 (10.74) ^***^	−0.022 (-17.03) ^***^
AUGE	2.392 (128.90) ^***^	0.870 (49.40) ^***^	0.509 (49.61) ^***^	0.166 (18.38) ^***^	0.124 (14.06) ^***^	0.071 (13.74) ^***^
Payment						
Pay	-	(base)	(base)	(base)	(base)	(base)
Sometimes pay	-	1.788 (115.52) ^***^	0.648 (71.84) ^***^	0.496 (62.58) ^***^	0.339 (43.80) ^***^	0.305 (66.99) ^***^
Do not pay	-	0.311 (33.78) ^***^	0.382 (71.15) ^***^	0.028 (5.95) ^***^	−0.185 (-40.25) ^***^	0.086 (31.94) ^***^
Other	-	−0.059 (-3.46) ^***^	0.075 (7.59) ^***^	−0.076 (-8.78) ^***^	−0.103 (-12.09) ^***^	0.045 (9.03) ^***^
No health care	-	−1.024 (-24.18) ^***^	−0.260 (-10.56) ^***^	−0.321 (-14.77) ^***^	−0.376 (-17.77) ^***^	−0.067 (-5.37) ^***^
Constant	- 2.292	1.315	0.409	0.775	0.124	0.215
Pseudo *R*^2^/ *R*^2^	0.081	0.064	0.080	0.027	0.022	0.016
Observations	1,554,227	581,048	581,048	581,048	581,048	581,048

Dependent variable for the logit model is a dichotomous indicator of whether a person has had health care during a particular year or not. Dependent variable for the OLS model is the number of health care use. t-score in parentheses ( ) and indicates significance level as follows: ***p ≤ 0.01, **0.01 < p ≤ 0.05, *0.05 < p ≤ 0.10. Confidence intervals are presented in brackets [ ].

In the logit model, most of the variables were statistically significant at 5 per cent p-value, mainly due to the large sample size of 1,554,227 individuals. However, in this study, we are more interested in evaluating practical significance; hence we want to estimate the size of the estimated coefficients and their impact on health care utilization. Also, we complement this study with individual models for the years 1992 to 2009 in order to assess which variables are important to predict use of health care services. The results suggest that the strongest predictors of health care use are the years the survey was conducted, gender, marital status, availability of electricity and water, insurance, type of housing, schooling, natural logarithm of income, and AUGE.

**Figure 1:**
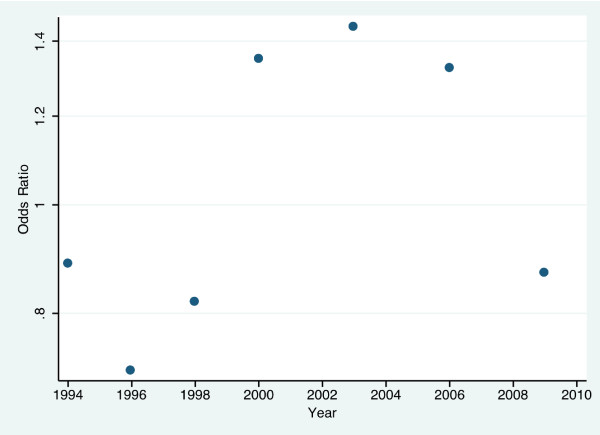
Odds ratios of health care use per year (base = 1992).

Results from the logit model estimation suggest that the odds of using health care throughout the years have changed over time. Between 1994 and 1998, the odds of using health care services were lower than in the base year 1992 (−0.121 and 95% CI (−0.138, -0.105) for 1994; -0.342 and 95% CI (−0.361, -0.323) for 1996; -0.201 and 95% CI (−0.218, -0.184) for 1998), whereas between 2000 and 2006 the odds of using health care services were higher than the odds for the reference year 1992 (0.300 and 95% CI (0.285, 0.314) for 2000; 0.366 and 95% CI (0.351, 0.380) for 2003; 0.281 and 95% CI (0.266, 0.297) for 2006), and then again we see that for 2009 the odds of using health care were 0.869 times the odds of using health care services in 1992 with a 95% CI of −0.156 to −0.124. We present an odds ratio of key variables derived from logit model estimation in Figure 1. This variation in the trend is accompanied by changes in policy and in the political structure of Chile. Chile in 1980’s brought policies that focused on increasing the private sector involvement in the country. The return to democracy in Chile was accompanied with a change in health policy. In 1990, the policies were oriented toward strengthening the public health sector through the injection of public resources, which improved the access to health care to the most disadvantaged groups [39]. In 1994, dissatisfaction with the public sector increased and called for need of new public resources to improve the provision of health care services [39]. In fact, in 1996 ISAPREs represented 31.5 per cent of the population covered by the health system [40]. After 2000, the Chilean health reform was intensified, and in 2005 the AUGE program was established [41].

Our results also suggest that females are more likely to use health care services than males. The odds of using health care services for females were 1.504 times the odds of males with a 95% confidence interval of 1.492 to 1.514 and a p-value lower than 0.001. Also, married people are more likely to use health care services than non-married, and our results suggest that the odds for married individuals are associated with 12.52 per cent higher than for non-married individuals (point estimate of 0.118, 95% CI (0.109, 0.126), and p ≤ 0.001). Further, there is also a positive association for those individuals who have portable water and electricity in their homes to use more health care services than the individuals who do not have these basic services (point estimate for water 0.147, 95% CI (0.132, 0.132), and p ≤ 0.001; and point estimate for electricity 0.153, 95% CI (0.134, 0.173), and p ≤ 0.001). Estimated results for the schooling variable are different from our expectation. We tend to expect that individuals with education will use more health care services than those individuals with no education, given the same health need. Our results, however, show that the odds of using health care services for those with education are only 0.304 times the odds of those without education, 95% CI (0.301, 0.308) and the p-value is less than 0.001. The estimated coefficients of income and AUGE also indicate that people with higher income have greater odds of using health care services (point estimate = 2.392, 95% CI (2.356, 2.428), and p ≤ 0.001). Likewise, being under the AUGE program increases the odds of health care use. It is worth noting that education and income usually result in higher use of health care services; however, educated persons tend to take a better care of their health and have fewer acute diseases (Muller, [42]). This may explain the relationships we found. Although health need variables were included as a control in our model, however, it could be these variables were not adequately measuring health needs, hence education might capture that differences, thus this estimated results.

In Table 3, we also present the results of 5 OLS models, which include: total health care use, preventive care, general practitioner visits, specialty visits, and emergency visits. Among the 5 different OLS models we find that school, AUGE, and type of payment are strongest predictors for utilization of health care services, preventive care, practitioners visits, specialty visits, and emergency visits. When comparing these 5 models for people with education we find that respondents with school education have 52 per cent lower probability of using health care services than those with no education (95% CI (−0.538, -0.501), and p ≤ 0.001). When analyzing for preventive services, practitioner visits, specialty visits, and emergency visits, we find that people with education were associated with 66.2 per cent lower probability of using preventive services than those without education (95% CI (−0.673, -0.651), p ≤ 0.001). At the same time, individuals with education are more likely to use general practitioner visits (4.8 per cent higher probability of use, 95% CI (0.038, 0.057)), specialty visits (5.5 per cent probability increase in the use of health care services with a confidence interval of 0.045 to 0.064), and emergency visits (4 per cent higher probability of use with a confidence interval of 0.034 to 0.045) compared to individuals with no education.

### Two-part model for the 2006 National Survey on Satisfaction and Out-of-Pocket payments

To provide further evidence of essential factors associated with health care utilization in Chile, we also analyzed the first National Survey on Satisfaction and Out-of-Pocket payments for the year 2006. Results of this estimation are presented in Table 4.

**Table 4:** **Estimation result of two**-**part model for health care utilization for the 2006 Satisfaction and Out**-**of**-**Pocket Payment Survey**

**Variable**	**Logit model**	**OLS (Health Care Use)**
Region:		
II	(reference)	(reference)
V	0.394 (1.38)	0.344 (1.00)
VIII	0.780 (2.77) ^***^	0.220 (0.64)
XIII	0.453 (1.73) ^*^	0.432 (1.33)
Gender	−0.006 (-0.03)	0.019 (0.08)
Age	0.015 (2.09) ^**^	−0.004 (-0.50)
Civil Status:		
Married	(reference)	(reference)
Living in-partner	0.262 (0.99)	0.071 (0.21)
Annulled/separated/divorce	0.351 (1.28)	0.119 (0.37)
Widowed	0.476 (1.80) ^*^	0.031 (0.10)
Single	0.259 (0.92)	0.142 (0.41)
School:		
No education	(reference)	(reference)
Elementary school	−0.087 (-0.17)	−0.420 (-0.82)
High school	−0.256 (-0.50)	−0.420 (-0.81)
Technical-professional school	−0.335 (-0.60)	−0.133 (-0.23)
Technical training center	−1.212 (-1.69) ^*^	−0.847 (-0.95)
Professional institute	−0.626 (-0.99)	−0.577 (-0.81)
University	−0.229 (-0.41)	−0.504 (-0.87)
Work status	0.029 (0.12)	−0.976 (-3.53) ^***^
Chronic Disease	0.799 (4.73) ^***^	0.022 (0.11)
Accident	0.528 (2.09) ^**^	0.387 (1.57)
Housing:		
House	(reference)	(reference)
Apartment	−0.011 (-0.04)	−0.114 (-0.42)
Tenement	0.581 (0.94)	−0.137 (-0.21)
Hut/shack	0.839 (1.24)	−0.616 (-0.78)
House:		
Own	(reference)	(reference)
Rent	−0.245 (-1.18)	0.129 (0.50)
Transfer	−0.178 (-0.70)	0.040 (0.13)
Insurance:		
Fonasa (group a/indigent)	(reference)	(reference)
Fonasa (group b)	0.708 (1.35)	−0.159 (-0.19)
Fonasa (group c)	0.467 (0.85)	−0.070 (-0.08)
Fonasa (group d)	0.860 (1.56)	−0.289 (-0.33)
Fonasa (do not know the group)	−0.325 (-0.55)	−0.617 (-0.67)
Isapre	0.584 (1.02)	−0.394 (-0.43)
Other system	0.814 (1.35)	−0.493 (-0.53)
None	−0.899 (-0.66)	−1.243 (-0.66)
Beneficiaries	0.029 (0.38)	−0.048 (-0.52)
Dependent	−0.227 (-1.05)	−0.142 (-0.56)
Emergency insurance	−0.181 (-0.47)	0.710 (1.69)
Additional insurance	−0.230 (-0.91)	−0.239 (-0.79)
Debt	0.190 (1.07)	−0.004 (-0.02)
Work insurance		
Yes	(reference)	(reference)
No	−0.547 (-2.86) ^***^	0.269 (1.14)
Do not work	−0.883 (-2.82) ^***^	−0.508 (-1.43)
Number of people in the household	0.098 (1.85) ^*^	0.083 (1.38)
Ln(OOP)	0.224 (12.01) ^***^	0.079 (3.17) ^***^
Ln(income)	−0.055 (-0.73)	0.129 (1.17)
Health care satisfaction:		
Very satisfied/satisfied	(reference)	(reference)
Indifferent	−0.465 (-2.26) ^**^	−0.102 (-0.40)
Very dissatisfied/dissatisfied	−0.238 (-1.22)	-.317 (1.39)
No answer	0.593 (0.92)	−0.400 (-0.38)
AUGE:		
No AUGE pathology	(reference)	(reference)
AUGE pathology	0.018 (0.08)	−0.248 (-1.07)
Do not know	0.087 (0.27)	0.276 (0.72)
No answer	−0.546 (-3.46) ^***^	−0.336 (-1.79)
Constant	- 3.032	0.634
Pseudo *R*^2^/ *R*^2^	0.258	0.133
Observations	1,388	470

Dependent variable for the logit model is a dichotomous indicator of whether a person has had health care utilization during a particular year or not. Dependent variable for the OLS model is frequency of health care use.

Z-score is presented in parentheses ( ) and indicates significance level is presented as followings: ***p ≤ 0.01, **0.01 < p ≤ 0.05, *0.05 < p ≤ 0.10.

For the logit model, estimated coefficients of the following variables were statistically significant at 5 per cent p-value: VIII region, XIII region, age, chronic disease, accident, work insurance, and the natural logarithm of out-of-pocket payment.

Our results indicate that the odds of using health care are 2.18 times higher for the VIII region (95% CI (1.255, 3.789), p-value = 0.006) and 1.57 times higher for the XIII region (95% CI (1.062, 2.627), p-value = 0.083) compared to that of the II region. Additionally, the odds of using health care services for individuals with chronic conditions are 122.33 per cent higher than the odds for individuals without chronic diseases (95% CI (1.597, 3.096), and p ≤ 0.001). Accidents increase the odds of using health care by 69.55 per cent with a 95% confidence interval of 1.035 to 2.779, with p-value equal to 0.036. The variable “work insurance” refers to welfare services or social security in the workplace and according to our results, having work insurance was positively associated with the odds of using health care, with p-value lower than 0.001. Finally, increases in the natural logarithm of out-of-pocket payment were associated with increasing the odds of using health care services by 25.11 per cent (95% CI (1.207, 1.298) and p ≤ 0.001). However, one needs to be cautious in interpreting this result because we used cross-sectional data, and the possibility of reverse causality, i.e. more utilization resulted in higher out-of-pocket payment, can exist.

For the second part of the model, estimated coefficients of the variables work status and natural logarithm of out-of-pocket payment were statistically significant. Therefore, having work is associated with a reduction of the use of health care services by 97.6 per cent (95% CI (−1.519, -0.433, and p ≤ 0.001). Additionally, as the natural logarithm of out-of-pocket payment increases by 1 per cent the frequency of use of health care services is associated with an increase of 1.082 per cent (95% CI (0.141, 1.137), p-value = 0.002). Interpretation of this result, however, should be cautious for the potential reverse causality. The relationship between types of health insurance and healthcare use was not statistically significant.

### Horizontal inequity

**Figure 2:**
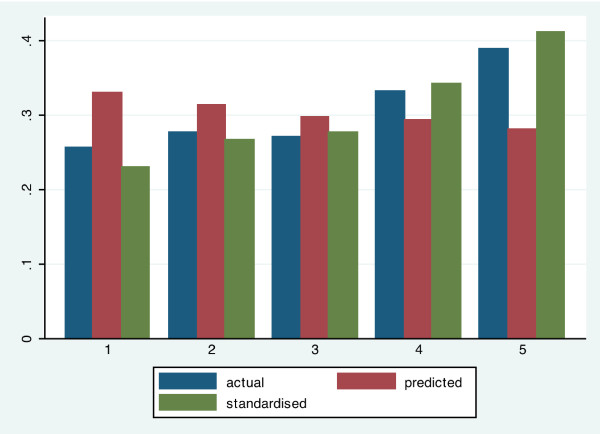
**Distribution of actual**, **predicted and standardized need for health care services by income quintile.**

The following section of this study includes an assessment of inequalities in the health care system using the 2006 Satisfaction and Out-of-Pocket Payment survey. We predicted needed medical care by demographic variables and also a list of eighteen health care problems (see Figure 2). Results of our estimation suggest pro-rich actual distribution with the poor having higher need-expected distribution. This is a result of the fact that “need,” as proxies for demographic and health problems, is more concentrated among the lower-income groups. After need-standardization, the wealthiest 20 per cent use medical care almost twice as much as the poorest 20 per cent.

Table 5 shows the distributions of actual need-predicted and need-standardized healthcare use for the year 2006.

**Table 5:** **Distributions of actual need**-**predicted and need**-**standardized health care use for year 2006**

**Probability of using medical care**							
	**Probit with controls**			**Need**-**standardized**			
				**With controls**		**Without controls**	
**Quintile**	**Actual**	**Need Predicted**	**Difference= predicted-actual**	**Probit**	**OLS**	**Probit**	**OLS**
Poorest 20%	0.256	0.330	−0.073	0.229	0.230	0.232	0.232
2^nd^ poorest 20%	0.276	0.313	0.037	0.265	0.266	0.267	0.267
Middle	0.271	0.297	0.026	0.277	0.276	0.276	0.276
2^nd^ richest 20%	0.332	0.293	0.040	.342	0.342	0.342	0.342
Richest 20%	0.389	0.281	0.108	0.411	0.410	0.408	0.408
Mean	0.305	0.303	0.002	0.305	0.305	0.305	0.305
Concentration	0.089	0.033		0.121	0.120	0.118	0.117
Index/HI_wv_							
Standard error	0.013	0.004		0.013	0.013	0.013	0.013
t-ratio	6.644	−8.028		9.469	9.404	9.234	9.219

Survey: Satisfaction and Out-of-Pocket Payment Survey (2006).

Table 5 supports this idea of a pro-rich actual distribution. The poorest 20 per cent used on average 7.3 per cent less health care services than the services they would be using according to their need, while the second wealthiest and the wealthiest 20 per cent used more health care services than their predicted need, 4 per cent and 10.8 per cent accordingly. After we standardized the values the wealthiest people used almost twice the health care as the poor people did.

We followed our analysis to decompose the effects of need factors and non-need factors in the utilization of health care services. Table 6 presents the main results.

**Table 6:** **Decomposition of concentration index for access to health care use**, **2006**

**Contributions to concentration index for any use of medical care**				
	** OLS**		**Probit partial effects**	
	**Absolute**	**Percentage**	**Absolute**	**Percentage**
**Need factors**				
Age-sex groups	−0.029	−32.95	−0.032	−36.36
Health problems	−0.003	3.409	−0.003	−3.409
Subtotal	0.032	36.36	−0.035	−39.77
**Non**-**need factors**				
Log household expenditure	0.102	115.91	0.111	126.14
Health insurance cover	0.008	9.091	0.009	10.22
Subtotal	0.110	125.00	0.120	136.36
Residual	0.010	11.36	0.003	3.41
Total	0.088			088
Horizontal inequity index	0.120			0.123

Survey: Satisfaction and Out-of-Pocket Payment Survey (2006).

The contribution of all need factors is negative, indicating that if utilization were determined by need alone, it would be pro-poor. The aggregate contribution of all need factors is about 36.36 per cent of the unstandardized index. Logarithm of household expenditures and health insurance coverage increases the concentration index by approximately 115.91 per cent and 9.09 per cent respectively.

The residual difference between the unstandardized concentration index and the sum of the contributions of all need and non-need factors is larger for the partial effects probit approach, mainly because this gives a slightly larger estimate of the contribution of household expenditure. Moreover, a positive horizontal inequity index indicates that the better-off make a greater use of health care services in Chile.

## Discussion

In this study we found evidence of inequities in health care utilization benefiting the better-off, despite the Chilean government’s new programs to promote equity in the use of health care services. The analysis of the distribution of actual need, predicted need and standardized need, and also the decomposition index all provide evidence of pro-rich inequities in the use of medical care. Moreover, our results indicate that the poor are using less health care services than expected according to their needs. This analysis of horizontal inequities in health care utilization supplement our two-part model analysis that focusing on variables affecting utilization of health care utilization. Such equity analysis produces important information for policy concern of equity in health care utilization, independent of two-part model analysis. Societies that concern equity of health care utilization will need to conduct similar equity analysis to provide evidence for policy assessment and recommendations.

The two-part model estimation indicated some key factors that are affecting utilization of health care services. We find that the major predictors of service utilization between 1992 and 2009 using the CASEN dataset are education, the implementation of AUGE program and the type of health care payment. This analysis provides important evidence of the achievements of the AUGE program, which, according to our results, increased Chilean's utilization of health care services. At the same time, while AUGE increased the overall utilization of health care services in Chile, it was still fell short of achieving equity. Our estimation in this study indicated that after AUGE was implemented, the utilization of health care in Chilean health care system is still pro-rich. That suggests either further policies are needed to improve equity of health care utilization in Chile, or the need to revise or modify AUGE in order to improve equity in health care utilization. Results from the analysis of the Satisfaction and Out-of-Pocket payments survey for the year 2006 suggest that the strongest predictors of health care use include work status and out-of-pocket payment. Results from these two surveys support that health care payment is an important variable to assess health care use. However, interpretation of the relationship between health care utilization and out-of-pocket payment should be cautious. As mentioned in the last section, due to our use of cross-sectional data, reverse causality could potentially exist. The estimated coefficient of the variable AUGE is not statistically significant for the year 2006; however, a possible explanation is that AUGE had just started in 2006. The relationship between types of health insurance and healthcare use is also not statistically significant, which suggest that Chileans’ utilization of health care may not be varied among different types of insurance.

In another study conducted in Chile analyzing the use of medical services, the authors concluded that AUGE reform was not necessarily improved equity in the use of health care services, and that there are still barriers to achieve the equitable use of health care services [43]. Also, in a study conducted by Vásquez, Paraje and Estay [44] consistent with our results, the authors used standardized concentration indices, found pro-rich inequity for specialized, dental, general practitioners and physician visits. They also found pro-poor distribution in the use of emergency room visits and hospitalizations.

### Limitations

There are several limitations of this study related to the use of secondary data. The first one is a longer self-reported recall period of one year or six months for most of the questions related to health care in the CASEN survey, which may increase recall bias. Also, we are aware that estimates of health care use can suffer from the same recall bias.

We also recognize that self-report bias might exist for variables such as service utilization and income. Individuals tend to under-report their income; which may lead to underestimation of inequalities across income groups. However service utilization can either be under or over reported, therefore, the present analysis may be biased but it is uncertain of the direction. Some researchers believe that self-reporting of physician visits may be unreliable [45]. Sometimes underreporting occurs in service utilization and is likely to increase as utilization of services increases [46]. If this is the case, then estimates of inequity can be underestimated in this study. Furthermore, in our model specification and estimation, there is potential bias as a result of omitted variables that were not included in the explanatory variables. While we did our best to specify the model that included all necessary variables based on theory and empirical evidence, as well as performing Hausman test, no model can ever be truly “complete” because of potential omitted variables not known to the researchers at the time of analysis, or due to data limitations. There are two possible omitted variables in our model estimation. The first one is availability of providers in community, which could be measured in terms of travel distance. Holding every other variables constant, availability of providers could have an impact on the utilization of health care. By omitting this variable, the potential bias is estimated coefficients of variables on insurance and household wealth (in our model, they are housing style and ownership, electricity, water, and disposal) could be over-estimated. At the same time, its direction may not be affected because it is expected that availability of providers will have the same direction of effect on the dependent variable as those explanatory variables mentioned above. The second possible omitted variable is alternative health care providers, such as traditional and complementary medicine providers, which were not measured in this study. The impact of this omitted variable is less certain. For utilization of alternative medicine providers could either be a complement or substitute to allopathic medicine (as was measured in this study), depending on the society and people. If Chileans who used alternative medicine providers as complement to allopathic medicine, then by omitting this variable we do not expect the sign of estimated coefficients on key explanatory variables to change, but the size might have been over-estimated. On the other hand, if Chileans who used alternative medicine providers as substitute to allopathic medicine, then by omitting this variable, not only the size of estimated coefficients may be over-estimated, but some of them may have the wrong direction.

Another limitation of this study is potential measurement errors in both dependent and explanatory variables that will affect both our model estimation and interpretations of the results. If there were measurement errors on the health status variables, it will affect the horizontal equity index estimation, and the estimated coefficients of explanatory variables in the two-part model could be over-estimated. If there was measurement error in the dependent variable, then the estimated coefficients of explanatory variables in the two-part model may not be accurate. Depending on the direction of measurement errors, estimated coefficients could be either over- or under-estimated.

In addition, because we used cross-sectional data for our analysis, there could be reverse causality exist between health care utilization and out-of-pocket payment; hence one has to be cautious in interpreting this result.

There were also missing values, especially from information regarding income that we needed to estimate before conducting the analysis. The large sample size of the data, however, may dampen the effect of negligible missing values.

### Policy implications

The findings of this study could help identify targets for policies improving equity in the use of health care in the Chilean health care system. In fact, policy-makers should assess why health care need is not being satisfied, at least for the first three quintiles of the population. The health care system is inequitable in the way that resources are distributed among income groups. Moreover, out-of-pocket payment is limiting the use of health care, which means the existing health insurance may not provide adequate financial access to health care. Hence, a new program such as AUGE with stronger financial support for Chileans could result in an improvement to a more equitable health care system. Its impact, however, will need to be further evaluated, as our results indicated that AUGE improved access to health care yet there are still inequity in health care utilization in Chilean health care system.

Finally, this analysis is also helpful to compare results of health care utilization with other countries in Latin America or countries that have a health care structure similar to the Chilean system. Such comparison may give readers and policy makers a better understanding of where Chile’s health care system stands in terms of equity in health care utilization. The approach of our analysis presented here could be replicated in other Latin American countries, as equity in health care has become a popular policy imperative in many countries. Furthermore, each country that undertakes a similar investigation may select additional policy variables that are important or unique to their country context. Accordingly, assessment results can provide relevant policy implication for improving its equity in health care utilization.

### Future research

A continuous, routine, and systematic assessment of equity in health care utilization over time may be helpful to understand the impact and implications of policies in the health care sector. Likewise, it is important to assess progress toward equitability in Chile in the area of health care utilization. Perhaps one important contribution this study made was the evidence related to the impact of AUGE; while it contributed to increasing the overall utilization of health care by Chileans, it also fell short of improving the equity of health care utilizations in Chile. A program like AUGE, with its intention to improve equity in health care will need regular evaluation, which can generate evidence-suggesting improvements in its policy objectives. Further, despite its policy intention to improve access and equity in health care, the reason it fell short calls for further investigation. Results of this further investigation will contribute to the advancement of equity in access to health care in Chile.

In terms of assessment method, disaggregated information from surveys by social groups is necessary, albeit not sufficient. Even though the available information is subject to some limitations for the study of equity problems in Chile, there is data that was collected but was not used or adequately analyzed. We expect to repeat this study in the near future and evaluate the progress of the health care system in the country as well as the impact of the reforms. Researchers in other Latin American countries where either the country has already implemented or is in the process of implementing health care policies to improve equity may want to do the same.

## Abbreviations

AUGE: Plan de Acceso Universal de Garantías Explícitas (Regime of Explicit Health Guarantees); CASEN: Encuesta de Caracterización Socioeconómica Nacional (National Socioeconomic Survey); FONASA: Fondo Nacional de Salud (National Health Fund); ISAPRE: Instituciones de Salud Previsional (Health Insurance Institutions).

## Competing interests

The authors declare that they have no competing interests.

## Authors’ contributions

ANM developed the original research idea and questions, obtained the data for this study, conducted data analysis, interpreted the results, and wrote the manuscript. CC contributed to the original research idea and questions, selection of statistical models for data analysis, interpreted the results, and contributed to the writing and revisions of the manuscript. All authors read and approved the final manuscript.
